# Arteriovenous Cerebral High Flow Shunts in Children: From Genotype to Phenotype

**DOI:** 10.3389/fped.2022.871565

**Published:** 2022-04-25

**Authors:** Berivan Tas, Daniele Starnoni, Stanislas Smajda, Alexandre J. Vivanti, Catherine Adamsbaum, Mélanie Eyries, Judith Melki, Marcel Tawk, Augustin Ozanne, Nicole Revencu, Florent Soubrier, Selima Siala, Miikka Vikkula, Kumaran Deiva, Guillaume Saliou

**Affiliations:** ^1^Department of Diagnostic Radiology and Interventional Radiology, Lausanne University Hospital (CHUV), Lausanne, Switzerland; ^2^Department of Neurosurgery, Centre Hospitalier Universitaire Vaudois, Lausanne, Switzerland; ^3^Department of Clinical Neurosciences, Lausanne University Hospital, Lausanne, Switzerland; ^4^de Duve Institute, Université Catholique de Louvain, Human Molecular Genetics, Brussels, Belgium; ^5^INSERM U1195 Petites Molécules de Neuroprotection, Neurogénération et Remyélinisation, Le Kremlin Bicêtre, France; ^6^Service de Radiologie Pédiatrique, Hôpital Bicêtre, Le Kremlin Bicêtre, France; ^7^Laboratoire Traitement et Communication de l'Information, TELECOM ParisTech, Paris, France; ^8^Department of Genetics, Hôpital Pitié-Salpêtrière, Paris, France; ^9^Department of Neuroradiology, Bicêtre Hospital, Le Kremlin Bicêtre, France; ^10^Service de Neuropédiatrie, Hôpital Bicêtre, Le Kremlin-Bicêtre, France; ^11^University of Lausanne, Lausanne, Switzerland

**Keywords:** vein of Galen aneurysmal malformation, cerebral arteriovenous fistula, arteriovenous shunt (AVS), Rendu-Osler-Weber disease, RASA1 mutation, EPHB4 mutation

## Abstract

**Objective:**

To study the genotypes and phenotypes of cerebral arteriovenous fistulas that drain or do not drain through the vein of Galen, and true vein of Galen aneurysmal malformations, in order to determine whether genotyping could help improve classification of these malformations and their management.

**Methods:**

We carried out a retrospective review of genetic and phenotypic data in databases of four centers. All children with cerebral arteriovenous fistula or vein of Galen aneurysmal malformations aged below 18 years at onset were included. We recorded the nature of the genetic variant or absence of variant, age at onset, type of malformation, symptoms at onset (hemorrhage, neurological deficit, hydrocephalus, incidental, and heart failure), type of venous drainage and the long-term outcome.

**Results:**

One hundred and fifteen children were included. Autosomal dominant variants were identified in 39% of patients. The most frequent variant affected was the *RASA1* gene (25%) followed by *EPHB4* (8%) and the HHT-associated genes (5%). HHT gene variants were only observed in pial arteriovenous fistula not draining into the vein of Galen; on the contrary, *EPHB4* variants were only seen in genuine vein of Galen aneurysmal malformation. *RASA1* variants were identified in all types of shunts.

**Conclusions:**

*EPHB4* variants seem specific to the vein of Galen aneurysmal malformation, *RASA1* variants are associated with either pial arteriovenous fistulas or with genuine VGAM and HHT gene variants seem specific to pial arteriovenous fistulas. The genetic data helps to classify these malformations and to guide treatment toward lowest risk of post-operative cerebral ischemic-hemorrhagic complications.

## Introduction

Recently, new genetic findings of pial fistulas and vein of Galen malformations have been described. Several published case series have identified Ephrin type-B receptor 4 (*EPHB4*) dominant variants in Capillary Malformation—Arteriovenous Malformation 2 (CM-AVM 2), especially in people with a vein of Galen aneurysmal malformation (VGAM) (1–3). These genetic variants are identified in ~10% of VGAMs. Other genetic variants described as frequently linked with cerebral arteriovenous fistulas and VGAMs include *RASA1* variants, associated with Capillary Malformation—Arteriovenous Malformation 1 (CM-AVM 1) or with Rendu-Osler-Weber disease (Hereditary Hemorrhagic Telangiectasia or HHT) (4–6). These findings open the possibility for targeted therapy in the treatment of arteriovenous malformations.

While extremely rare, VGAM is the most frequent high-flow vascular malformation in babies of all ages combined. It is lethal in almost 100% of cases if left untreated. While endovascular embolization has clearly improved the outcome, several recent case series (7, 8) have shown that the long-term prognosis in this malformation is more severe than the usually described medium-term prognosis (9–11). On the other hand, the true aneurysmal malformations of the vein of Galen are distinguished from the pial and/or choroidal arteriovenous shunts that drain into the vein of Galen by the type of venous drainage. Drainage is abnormal in true VGAM, and the vein of Galen (actually the embryonic venous precursor) drains only the shunt; the deep drainage of the normal brain occurring by alternative venous routes, in particular *via* the latero-mesencephalic and latero-pontic veins toward the higher petrous sinuses (12). For choroidal or pial arteriovenous fistulas flowing into the Galen vein, the abnormal and normal venous drainage are however, in competition. In our experience, especially in babies, it is often difficult to differentiate an authentic aneurysmal malformation of the vein of Galen from arteriovenous shunts draining into the vein of Galen. Moreover, because these defects are rare, malformations draining into the vein of Galen or the nearby venous system are frequently described in the literature as genuine aneurysmal malformation of the vein of Galen, without specifying the characteristics of the veins draining the shunt.

The endovascular occlusion of the vein of Galen in VGAM theoretically allows the malformation to be occluded. However, it exposes the child to severe hemorrhagic or ischemic venous complications from any arteriovenous shunts in competition with the deep cerebral venous system if it is not a true VGAM. Our aim here is to describe the genotypes and phenotypes of pial fistulas feeding or not feeding the vein of Galen and true VGAMs, in order to determine whether knowledge of the genotype would help to improve the classification of these two distinct malformations; thereby leading to better management.

## Materials and Methods

We carried out a retrospective review of genetic and phenotypic cerebral arteriovenous shunt databases of four centers: the Inserm Research Unit of the Bicêtre hospital (UMR-1195), Kremlin Bicêtre, France, the Genetics Department of the Pitié-Salpêtrière hospital, Paris, France, the Human Molecular Genetics laboratory (GEHU) of the Duve Institute, Catholic University of Louvain, Brussels, Belgium and the department of neuroradiology, Lausanne University Hospital (CHUV), Lausanne, Switzerland. We screened all patients having at least one of the following diseases: hereditary hemorrhagic telangiectasia [HHT 1 (OMIM 187300), 2 (OMIM 600376), 3 (also called Juvenile Polyposis/HHT, OMIM 175050)], capillary malformation—arteriovenous malformation 1 (CM-AVM 1, OMIM 608354) or capillary malformation—arteriovenous malformation 2 (CM-AVM 2, OMIM 600011). Genetic results used in the present study have been published previously (3–6, 13).

**Figure 1:**
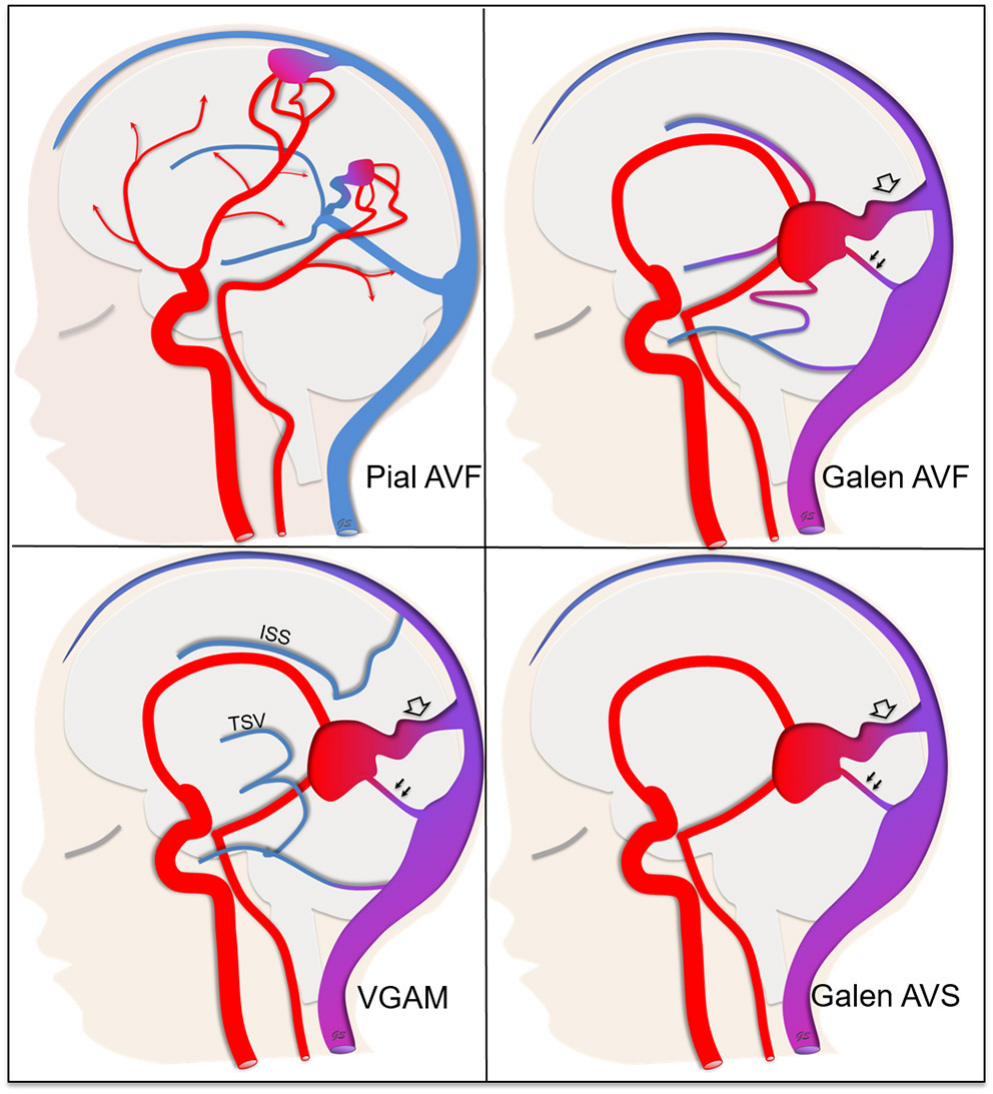
Classification of arteriovenous shunts into four sub-types. **Pial AVF** (pial arteriovenous fistula) not draining directly into the vein of Galen. The deep venous drainage is anatomically normal. **Galen AVF** (Galen arteriovenous fistula) draining directly into the vein of Galen and either a pial or choroidal AVF. The deep venous drainage is anatomically normal, that means that the shunt can be responsible for venous reflux through the deep venous drainage (single arrows). **VGAM** (Genuine Vein of Galen aneurysmal malformation) draining directly into the vein of Galen. The deep venous system is disconnected from the vein of Galen itself with the deep cerebral veins [especially thalamostriate veins (TSV) and inferior sagittal sinus (ISS)] draining *via* alternative pathways either through the falcorial sinuses or lateropontic and lateromesencephalic veins to the superior petrosal sinus. That means that the shunt can't be responsible for venous reflux through the deep venous system. **Galen AVS** (Galen arteriovenous-shunt) draining into the vein of Galen but with unknown anatomy of the deep cerebral veins. For shunts that drain directly into the vein of Galen, we noted the presence of a straight sinus (double arrow) and/or a falcine sinus (empty arrow).

All children with a cerebral arteriovenous fistula or a VGAM aged <18 years at onset were included. A true VGAM corresponded to a choroidal arteriovenous shunt draining directly into the embryonic precursor of the vein of Galen with normal deep cerebral venous drainage *via* alternative venous delivery routes, and without identifiable connection between the vascular malformation and the deep venous drainage on angiography, or on MRI or MRA. Cerebral (pial or choroidal) arteriovenous fistulae flowing directly into the vein of Galen together with the deep venous drainage of the brain were considered as Galen arteriovenous fistulae (Galen AVFs). Arteriovenous shunts draining directly into the vein of Galen without indication of whether or not the deep venous drainage of the brain flows *via* alternative drainage pathways were considered as Galen AV-shunts. The latter could be either a VGAM or an AVF, feeding into the vein of Galen. We thus classified malformations into four sub-types: non-galenic cerebral arteriovenous fistulas that belong to pial AVFs (called pAVF), AV-fistulas that drain directly into the vein of Galen, which are either pial or choroidal AVFs (called Galen AVF), Galen AV-shunts (called Galen AVS) or genuine VGAMs (called VGAM) (Figure 1).

We analyzed the following data: type of genetic variant or absence of genetic variant, age at onset, type of malformation, symptoms at onset (hemorrhage, neurological deficit, hydrocephalus, incidental, and heart failure), type of venous drainage at onset and long-term outcome. For arteriovenous shunts draining into the vein of Galen, we noted the presence or lack of a remaining embryonic venous sinus with a falcine sinus and/or the mature straight sinus. We defined the straight sinus as the sinus communicating with the vein of Galen and the torcular, and located along the junction of the tent of the cerebellum and the falx cerebri. We considered the sinuses communicating with the vein of Galen and the torcular, but located only in the falx cerebri remote from the junction of the tent of the cerebellum as falcine sinuses. We assessed long-term outcome by the pediatric modified Rankin Scale (14). We considered mRS 0–2 as a good evolution and mRS 3–6 as a poor evolution. In the event of termination of pregnancy, mRS was classified as 6.

### Statistical Analysis

We used descriptive statistics to represent the distribution of clinical and genetic variables studied in our population. Variables are presented as medians with interquartile range (IQR).

We performed analyses using the Pearson's Chi-squared test with Yates' continuity correction if required, for categorical variables. We performed statistical analyses using python language (Python Software Foundation. Python Language Reference, version 2.7. Available at http://www.python.org) with “SciPy” modules (12). Margins of error were expressed as the 95% confidence interval. The statistical significance level for all tests was set at 0.05. Due to the small sample size and as variables were not independent, we did not carry out a multivariable analysis.

## Results

One hundred and fifteen pediatric patients were included in this series. Of these, 38 had a pAVF, 8 a Galen AVF, 5 a Galen AVS, and 64 a VGAM. The clinical follow-up was available in 86 patients (75%), with 9 patients having a clinical follow-up of <1 month due to termination of pregnancy or death within a month after birth. The median clinical follow-up for the 77 patients still alive 1 month after birth was 27 months (IQR: 8–52). The clinical outcomes and genetic findings are displayed in Table 1. Combining all patients, the final clinical outcome was good in 62% and poor in 38%.

**Table 1:** Number of pathogenic variants found in the four types of arteriovenous malformations and patient outcome at the last clinical follow-up.

	***n***	**m/f (MD)**	***RASA1***	***EPHB4***	**HHT-associated gene variant**	No variant or other variant^**^	**Good/poor outcome % (MD)**	**Median FU time (IQR) (MD)**
**pAVF**	38	58/42% (2)	19	0	6	13	79/21% (5)	12 months (5–28), (6)
**Galen AVF**	8	38/63% (0)	3	0	0	5	100/0% (5)	4.5 months (2–8) (0)
**Galen AVS**	5	25/75% (1)	5	0	0	0	0/100% (3)	NA^*^
**VGAM**	64	48/52% (3)	2	9	0	53	51/49% (11)	42 months (24–53) (16)

*m, means male; f, female; NA, not applicable; FU, follow-up; MD, missing data*.

* *Of the 5 Galen AVS patients, the outcome was only available in 2 (Poor: 1 died after birth and the second at 156 months)*.

** *Only one patient had another identified variant (GLMN gene)*.

A germline variant was identified in 38% of patients. This percentage was 70% in the population harboring at least one cutaneous capillary malformation or mucocutaneous telangiectasia, and 16% in the population without a cutaneous capillary malformation. Results of genetic testing of the overall population are shown in Supplementary Table 1.

The most frequent genetic variant affected *RASA1* (25%), followed by *EPHB4* (8%) and two HHT gene variants [5%; *ENG* variant (5 patients) and *ALK1* variant (1 patient) and no other HHT gene variants]. One patient had another variant in the *GLMN* gene encoding Glomulin/FKBP Associated Protein, essential for normal development of the vasculature (15). Of the six HHT-associated gene variants, the variant was transmitted in four patients and *de-novo* in one (missing data (MD) in one). For six of the nine patients carrying *EPHB4* heterozygous variants, the variant was inherited. Finally, in the 29 *RASA1* variants, the variant was transmitted in 13 patients, *de-novo* in eight patients and unknown in two patients.

The HHT gene variants were only observed in pial AV-fistulas. On the contrary, *EPHB4* variants were only seen in VGAM. *RASA1* variants were identified in all four malformation subtypes. However, *RASA1* was more frequently associated with AVF (all three types including pAVF, Galen AVshunt, and Galen AV fistula) than with true VGAM (*p* < 0.0001).

Regarding the nature of venous drainage and particularly, a remaining embryonic falcine sinus, we did not identify a relationship with genetic status. However, among patients with a genetic variant, a falcine sinus was only observed in *EPHB4* variants (*p* = value not significant).

The main findings concerning a correlation between the phenotype (symptoms at onset, outcomes and skin abnormalities) and genotype are shown in Table 2.

**Table 2:** Clinical data of patients at presentation and phenotype-genotype correlation, with *p*-values.

	**HHT-associated gene (p value)**	**RASA1 (p value)**	**EPBH4 (p value)**	**No variant or other variant (p value)**	***Overall p-value***
**Shunt with drainage through the vein of Galen N/Y/NR**	6^*^/0/0 (NA)	23/6/0 (p <0.00001)	0/9/0 (NA)	18/53/0 (p <0.0001)	NA
**Gender M/F/NR**	1/5/0 (NS)	14/12/3 (NS)	3/4/2 (NS)	36/34/1 (NS)	0.41
**Among VGAM: presence of a straight sinus N/Y/NR**	none (NA)	0/1/5 (NA)	4/2/3 (NS)	19/26/8 (NS)	0.46
**Among VGAM: presence of a falcine sinus N/Y/NR**	none (NA)	1/0/5 (NA)	1/5/3 (NS)	21/24/8 (NS)	0.31
**Outcome good/poor/NR**	6/0/0 (NA)	16/6/7 (NS)	4/5/0 (NS)	34/25/12 (NS)	NA
**Prenatal diagnosis Y/N/NR**	1/5/0 (NS)	6/21/2	5/4/0	30/28/13	0.034
**Hydrocephalus or macrocrania at onset Y/N/NR**	0/6/0 (NA)	5/21/2 (NS)	2/7/0 (NS)	12/54/5 (NS)	NA
**Neurological deficit or seizure at onset Y/N/NR**	5/1/0 (<0.0001)	7/19/2 (0.071)	0/9/0 (NA)	3/61/7 (0.001)	NA
**Hemorrhage at onset Y/N/NR**	2/4/0 (0.034)	2/23/3 (NS)	0/9/0 (NA)	2/64/5 (NS)	NA
**Cardiac failure at onset Y/N/NR**	0/6/0 (NA)	9/18/2 (0.046)	6/3/0 (NS)	40/28/3 (NS)	NA

*NS, not significant; NA, not applicable; NR, not reported; N, no; Y, yes; M, male; F, female*.

* *among HHT-associated genes, an ENG variant was observed in 5 patients and an ALK1 variant in 1 patient*.

## Discussion

The central findings of the present case series are that *EPHB4* heterozygous variants were exclusive to VGAM and HHT-associated variants exclusive to AVF, especially those that do not drain through the vein of Galen. Further, although present in all four malformation sub-type groups, *RASA1* variants were significantly associated with the three other sub-types of AVF rather than VGAM.

Combining all four types of shunts, a genetic variant was identified in more than 1/3 of cases. This percentage increased to 70% in the population harboring cutaneous capillary malformation or mucocutaneous telangiectasia. It was also quite high in the population without cutaneous capillary malformation, as 16% of patients had a genetic variant. We should note that mucocutaneous telangiectasias may be absent at younger age and only appear later. Since all variants show dominant inheritance with a 50% risk of transmission to the offspring and possible affection of multiple organs, genetic screening of *EPHB4, RASA1*, and the three HHT-associated genes has to be considered for a high-flow intracerebral vascular malformation, even if the patient does not have evocative skin abnormalities. Moreover, if a *RASA1* variant is present in a mosaic state, the patient might not have skin lesions, or detectable germline variants (16).

Regarding the venous anatomy of VGAM, Raybaud et al. (17) first described that the deep venous collector was the medial vein of the prosencephalon, the embryonic precursor of the vein of Galen. Later, Lasjaunias et al. (18) reported that in VGAM, the ectatic venous pouch “freezes” the normal venous maturation at the 3–4-month embryonic stage and then characterized the possible alternative venous routes disconnected from the vein of Galen in VGAM. They finally concluded that VGAMs are likely the earliest AV shunts occurring *in utero*, before the end of the cerebral sino-venous maturation. As these malformations are extremely rare, arteriovenous shunts draining into the vein of Galen or the nearby venous system are frequently described in the literature as genuine aneurysmal malformations of the vein of Galen, without specifying the route of the deep venous drainage. Misclassifications of AVFs or arteriovenous malformations that drain into a dilated vein of Galen as true VGAMs are also frequent (19). For example, as already underlined (2), it is very unlikely that the malformation presented in the case report of Chida et al. (20) is an authentic VGAM. It is probably a pial fistula that drains into the upper vermian venous system. The same is true for patients 1 and 3 in another case series of presumably vein of Galen aneurysmal malformations (21). In patient 1, the shunt is located in the torcular, and the arterial feeders belong to the dural branches, suggesting a dural sinus malformation. For patient 3, the shunt is located in the choroidal fissure and drains secondarily into the vein of Galen, and is likely a choroidal fistula. In a further case series on dural arteriovenous shunts (22), the authors specified in their Figure 3 that the vein of Galen is dilated; however, it seems very probable that the torcular is dilated while the vein of Galen itself is not visible. All these example case series have an abnormal anatomy in common, directly or indirectly implicating the Galen vein or nearby dilated veins. However, they do not constitute authentic VGAMs. The data indicate that classification is difficult especially if physicians do not deal routinely with these diseases, as is the case in most neurovascular centers around the world. Even recently in a highly-experienced center, authors described that in their previously classified VGAMs, internal cerebral vein communication with the VGAM is not uncommon and requires dedicated pre-procedural imaging to identify it (23).

Accurate classification is crucial due to the therapeutic implications, which differ between a true VGAM and a pial fistula that drains into the vein of Galen. In theory, if we completely occlude the vein of Galen in a VGAM; as the deep venous system is disconnected, such treatment of a pial fistula might lead to deep venous ischemic or hemorrhagic complications due to competing venous flows from the malformation and the cerebral deep venous system. In this respect, genetic findings could be very useful to guide correct classification. Here, we observed that *EPHB4* genetic variants seem specific for VGAM, which is not the case for *RASA1* variants or HHT-associated gene variants. In cases of no determined genetic variant or *RASA1/*HHT-gene variants, we need to thoroughly define the deep venous drainage to classify the shunt correctly as a VGAM, or rather a pial or choroidal fistula draining into the vein of Galen. As the *EPHB4* variant appears specific for VGAM, for patients with this variant it seems feasible to consider a treatment that completely thromboses the vein of Galen, thereby definitively occluding the malformation while limiting the risks of deep vein complications.

We hypothesize several reasons why *EPHB4* variants might be specific for VGAM. First, this genetic variant is peculiar to arteriovenous damage in this anatomical location. Transgenic zebrafish embryos injected with the antisense morpholino, ephb4a-MO exhibited marked vascular anomalies of the dorsal cranial vessels including both dorsal longitudinal and mesencephalic veins, while the upper and lower middle layers of the cranial vessels did not reveal an abnormal vascular pattern (3). In addition, *RASA1* or *EPHB4* deficiency in zebrafish induced similar abnormalities in blood vessel formation and function (24), and phenotypes in mice with *Rasa1* variants are relatively restricted to the blood vessel compartment and not limited to the midline (25). In transgenic mouse models of HHT, the whole-body vasculature, especially post-capillary venules may be affected and the phenotype is not limited to the midline vasculature (26, 27). Second, genetic variants of *EPHB4* may be expressed very early in embryogenesis as suggested by Lasjaunias et al. (18), which would explain the persistence of the embryonic venous anatomy in VGAM and lack of maturation to the adult anatomy. This correlates with our findings that prenatally-discovered malformations were less frequently linked with HHT-associated variants or *RASA1* variants (*p* = 0.034). A likely reason is that most AV-shunts caused by HHT-associated or *RASA1* variants are related to cortical cerebral fistulas, difficult to identify in prenatal imaging, especially by ultrasound, compared to deep cerebral AV-shunts with large dilatations of the venous drainage. This supposed early expression of the *EPHB4* variant was also correlated with a higher risk of high-flow cardiac failure. The risk was lower in cases of HHT-associated or *RASA1* variants, which suggests that these shunts appear later on during embryogenesis. Finally, the fact that HHT-associated and *RASA1*/*EPHB4* genes belong to two different signaling pathways may play a role. Indeed, loss-of-function variants in *EPHB4* and *RASA1* affect the EPHB4-RAS-ERK signaling pathway (1) and gene variants in HHT, the TGF-β/BMP signaling pathway (28) with molecular differences between all the proteins. The two pathways may be activated at different time points in development and control distinct developmental aspects, which may be a reason we identified variants in *RASA1/EPHB4* genes in vein of Galen aneurysmal malformations with a specific ratio, but not variants in HHT-associated genes in our present series. This requires additional data to properly understand the underlying mechanisms.

Regarding the mode of presentation, as previously noted (6) our case series confirms the higher risk of bleeding in AVFs related to HHT-associated variants (*p* = 0.034) and the risk of neurological deficit or seizure at onset (*p* < 0.0001) (Table 2). Locoregional venous hypertension associated with cerebral arteriovenous malformations is likely to lead to an epileptic seizure or neurological deficit due to vascular steal or chronic venous ischemia in all types of malformations. However, in our experience (unpublished data), and in the literature, HHT-associated variants are frequently linked to cerebral parenchymal malformations, especially polymicrogyria (28–30). One suggestion to explain the malformations associated with HHT-associated variants is aberrant hypersprouting angiogenesis during corticogenesis, leading to the production of polymicrogyria (29). To us, the double impairment of cerebral malformation and venous hypertension could explain the more frequent association of HHT with epilepsy or neurological deficit.

Although this series is the largest published to date on this topic, due to its retrospective nature and the limited data, our findings need to be supported by additional series. In particular, additional cohorts that meticulously detail the anatomy of the deep venous system in arteriovenous shunts draining into the vein of Galen. This will improve our knowledge of these rare but potentially serious pediatric diseases and seems warranted.

## Conclusion

*EPHB4* genetic variants seem specific to true VGAM. On the other hand, *RASA1* variants seem associated with either pial or choroidal fistulas or with VGAM, particularly for shunts that drain into the vein of Galen. HHT-associated variants appear to be specifically related to pial fistulas. These data help to improve classification of malformation types and thereby, in choosing the appropriate treatment. Occlusion of the venous collector of the shunt would cure the malformation in the case of a VGAM associated with an *EPHB4* variant. However, we should pay special attention to the venous anatomy of arteriovenous shunts draining into the vein of Galen associated with *RASA1* variants due to the possibility of competing abnormal venous drainage and cerebral deep venous drainage, generating a risk of post-operative cerebral ischemic-hemorrhagic complications in cases of occlusion of the venous collector of the malformation.

## Data Availability Statement

The datasets presented in this study can be found in online repositories. The names of the repository/repositories and accession number(s) can be found below: https://databases.lovd.nl/shared/variants/EPHB4/unique#object_id=VariantOnTranscriptUnique%2CVariantOnGenome&id=EPHB4o=owned_by_%2CASC&search_transcriptid=00007167&page_size=100&page=1.

## Ethics Statement

Written inform consent to participate in medical studies was provided by the participant's legal guardian/next of kin.

## Author Contributions

BT, DS, and GS: conceptualized and designed the study, designed the data collection instruments, collected data, carried out the initial analyses, drafted the initial manuscript, and reviewed and revised the manuscript. SSm, AV, CA, ME, JM, MT, AO, NR, SSi, FS, MV, and KD: collected data, carried out the initial analyses, and reviewed and revised the manuscript. All authors approved the final manuscript as submitted and agree to be accountable for all aspects of the work.

## Funding

The earlier genetic studies of some of the VGAM patients were supported by a grant from the Agence de Biomedecine (2015) and Inserm (JM). The earlier genotyping studies of some of the patients were supported by the Fonds de la Recherche Scientifique–FNRS Grants T.0026.14 and T.0247.19 (to MV). Open access funding provided by University of Lausanne.

## Conflict of Interest

The authors declare that the research was conducted in the absence of any commercial or financial relationships that could be construed as a potential conflict of interest.

## Publisher's Note

All claims expressed in this article are solely those of the authors and do not necessarily represent those of their affiliated organizations, or those of the publisher, the editors and the reviewers. Any product that may be evaluated in this article, or claim that may be made by its manufacturer, is not guaranteed or endorsed by the publisher.
