# Supplementary material for: Arteriovenous Cerebral High Flow Shunts in Children: From Genotype to Phenotype
Source: Front Pediatr. 2022 Apr 25;10:871565. doi: 10.3389/fped.2022.871565 (PMC9081809; doi:10.3389/fped.2022.871565)
Supplement: Supplementary file 1 [file Table_1.docx]

| patient number | Type of shunt | Gender | single vs mutliple shunts | genetic variant | amino acid | coding variant | Transmission |
| --- | --- | --- | --- | --- | --- | --- | --- |
| 4 | Pial AVF | f | single | RASA1 | p.Asp532Glyfs*2 | c.1594dup | de novo |
| 5 | Pial AVF | f | single | RASA1 | p.Arg398X | c.1192C>T | Transmitted |
| 6 | Pial AVF | M | single | RASA1 | p.Y164X | c.492C>G | Transmitted |
| 7 | Pial AVF | F | single | GLMN |  | c.1214+2T>C | unknown |
| 16 | Pial AVF | f | single | ENG | p.Asn121Profs*47 | c.523G>T | de novo |
| 17 | Pial AVF | f | single | ENG | p.Phe389Cys | c.1166T>G | Transmitted |
| 18 | Pial AVF | f | multiple | ENG | p.His219Argfs*10 | c.636_655dup | Transmitted |
| 19 | Pial AVF | f | multiple | ALK1 | p.Ser305Phe | c.914C>T | unknown |
| 20 | Pial AVF | m | single | ENG | p.Val49Phe | c.145G>T | Transmitted |
| 21 | Pial AVF | f | single | ENG |  | c.360+1G>A | Transmitted |
| 22 | Pial AVF | m | multiple | RASA1 | p.Leu186Glufs*3 | c.556_562del | de novo |
| 23 | Pial AVF | m | single | RASA1 | p.Arg903* | c.2707C>T | Transmitted |
| 24 | Pial AVF | f | single | RASA1 | p.Gln503Hisfs*17 | c.1509del | de novo |
| 25 | Pial AVF | m | single | RASA1 |  | c.1102+2T>C | de novo |
| 26 | Pial AVF | f | single | RASA1 | p.Gly89Argfs*22 | c.261_262del | Transmitted |
| 27 | Pial AVF | m | multiple | RASA1 | p.Gln808* | c.2422C>T | Transmitted |
| 28 | Pial AVF | m | single | RASA1 | p.Ser523Cysfs*9 | c.1567_1568del | de novo |
| 29 | Pial AVF | m | single | RASA1 | p.Leu128Argfs*29 | c.383_384del | unknown |
| 30 | Pial AVF | m | single | RASA1 | p.Leu814X + p.Ala99Val | c.2441del, c.296C>T | Transmitted |
| 31 | Pial AVF | f |  | RASA1 | p.Leu159Glyfs*20 | c.475_476del | Transmitted |
| 32 | Pial AVF | m | single | RASA1 | Splicing | c.828+3A>T | de novo |
| 34 | Pial AVF |  | multiple | RASA1 |  |  | unknown |
| 35 | Pial AVF |  | single | RASA1 | p.Arg903X | c.2707C>T | Transmitted |
| 36 | Pial AVF | F | single | RASA1 |  |  | unknown |
| 37 | Pial AVF | M | single | RASA1 |  |  | Transmitted |
| 38 | galen AVF | f | single | RASA1 |  |  | unknown |
| 43 | galen AVF | m | single | RASA1 | p.Asn838Lysfs*2 | c.2513dup | Transmitted |
| 44 | galen AVF | m | single | RASA1 | p.Gly487Glufs*11 | c.1455del | unknown |
| 45 | galen AVF | f | single | RASA1 | p.Leu902Phefs*9 | c.2703del | de novo |
| 68 | VGAM | m | single | EPHB4 | p.Val870Glu | c.2609T>A | Transmitted |
| 69 | VGAM | F | single | EPHB4 | p.E664K | c.1990G>A | unknown |
| 70 | VGAM |  | single | EPHB4 |  |  | unknown |
| 71 | VGAM | F | single | EPHB4 | p.R150Efs*74 | c.447_448insGAAG | unknown |
| 72 | VGAM |  | single | EPHB4 | p.Val330MET | c.988G>A | Transmitted |
| 73 | VGAM | F | single | RASA1 | p.R512X | c.1534C>T | Transmitted |
| 96 | VGAM | m | single | EPHB4 | p.Met814_Val829del | c.2484+1G>T | Transmitted |
| 97 | VGAM | f | single | EPHB4 | p.Hisl91Alafs*32 | c.570dupG | Transmitted |
| 98 | VGAM | m | single | EPHB4 | Splicing | c.2484+2insT | Transmitted |
| 99 | VGAM | f | single | EPHB4 | p.Cys107Arg | c.319T>C | Transmitted |
| 110 | VGAM | m | single | RASA1 | p.Met306Thr | c.917T>C | unknown |
| 111 | galen AVS |  |  | RASA1 | p.Arg709X | c.2125C>T | Unsure |
| 112 | galen AVS | f | single | RASA1 | p.Arg993Valfs*3 | c.2977del | Transmitted |
| 113 | galen AVS | f | single | RASA1 | p.Glu1008Aspfs*16 | c.3024del | Transmitted |
| 114 | galen AVS | m |  | RASA1 | p.Glu763Val | c.2288A>T | Unsure |
| 115 | galen AVS | f | single | RASA1 | p.Leu845Thrfs*38 | c.2532_2536delTT AA | de novo |
